# Trait Aggression is Reflected by a Lower Temporal Stability of EEG Resting Networks

**DOI:** 10.1007/s10548-022-00929-6

**Published:** 2022-11-18

**Authors:** Tobias Kleinert, Kyle Nash

**Affiliations:** 1 Department of Psychology, University of Alberta, Edmonton, AB T6G 2E9 Canada; 2 Department of Ergonomics, Leibniz Research Centre for Working Environment and Human Factors, Ardeystr. 67, 44139 Dortmund, Germany

**Keywords:** Aggression, EEG, Microstates, Neural traits, Resting-state

## Abstract

Trait aggression can lead to catastrophic consequences for individuals and society. However, it remains unclear how aggressive people differ from others regarding basic, task-independent brain characteristics. We used EEG microstate analysis to investigate how the temporal organization of neural resting networks might help explain inter-individual differences in aggression. Microstates represent whole-brain networks, which are stable for short timeframes (40–120 ms) before quickly transitioning into other microstate types. Recent research demonstrates that the general temporal stability of microstates across types predicts higher levels of self-control and inhibitory control, and lower levels of risk-taking preferences. Given that these outcomes are inversely related to aggression, we investigated whether microstate stability at rest would predict lower levels of trait aggression. As males show higher levels of aggression than females, and males and females express aggression differently, we also tested for possible gender-differences. As hypothesized, people with higher levels of trait aggression showed lower microstate stability. This effect was moderated by gender, with men showing stronger associations compared to women. These findings support the notion that temporal dynamics of sub-second resting networks predict complex human traits. Furthermore, they provide initial indications of gender-differences in the functional significance of EEG microstates.

**Supplementary Information:**

The online version contains supplementary material available at 10.1007/s10548-022-00929-6.

## Introduction

Aggression is widely regarded as a maladaptive trait in modern societies, as it leads to negative consequences for both victims and aggressors. For example, trait aggression predicts domestic violence (Ruddle et al. 2017), aggression in the workplace (Douglas and Martinko 2001), and aggressive driving behavior (Deffenbacher et al. 2001). On the other hand, trait aggression is associated with adverse outcomes for aggressors, including social exclusion (Twenge et al. 2001), mental health issues (Blair 2001; Twenge and Campbell 2003), and imprisonment (Falk et al. 2017). Interestingly, there are large inter-individual differences in trait aggression (for reviews, see Wilkowski and Robinson 2008; DeWall et al. 2011a), with only a small percentage of the population accounting for the majority of violent acts (Falk et al. 2014). Although aggression has been extensively studied on social (e.g., Bandura 1973; Berkowitz 1993), psychological (e.g., Anderson and Bushman 2002), and neuroscientific (e.g., Nelson and Trainor 2007) levels, it remains unclear if and how aggressive people differ from others in regard to fundamental, task-independent, brain characteristics.

Here, we follow a neural trait approach to examine how basic neural processing at rest may help explain the sources of individual levels of aggression. The neural trait approach involves measuring stable characteristics in resting-state brain recordings and investigating whether these characteristics are associated with cognitive, biological, and behavioral processes (e.g., Schiller et al. 2014, 2020a, b, 2021; Leota et al. 2021; Nash et al. 2022; Kleinert et al. 2022). This task-independent approach compliments existing, task-dependent analyses of neural and psychological processes (for more on the benefits of task-independence, see Berkman and Falk 2013). Perhaps most importantly for individual difference research, neural traits are objective—unadulterated by personal biases and demand characteristics.

Research examining resting-state electroencephalography (EEG) has demonstrated that neural network activation remains stable for approximately 40–120 ms before quickly transitioning into another network type. These “basic building blocks of cognition” (Lehmann et al. 1998) are called *microstates* (for reviews, see Khanna et al. 2015; Michel and Koenig 2018). During spontaneous neural processing at rest, only four microstate types (A, B, C and D) typically explain 70 to 80% of variance in individual EEGs (Koenig et al. 2002). All four types represent widely distributed networks, which share common neural generators including the anterior cingulate cortex (ACC), the insula, and parts of the parietal and prefrontal cortices (Custo et al. 2017; Milz et al. 2017), and resemble fMRI resting networks (Britz et al. 2010; Musso et al. 2010; Yuan et al. 2012; Abreu et al. 2021). Moreover, durations and occurrences of the four different microstate types are highly correlated (average correlation of durations: *r* = 0.79; average correlation of occurrences: *r* = 0.51; Khanna et al. 2014), supporting the notion that the general *microstate stability* represents a neural trait that overarches characteristics of specific microstate types. Interestingly, there is a high degree of heterogeneity in microstate stability at rest, across all microstate types (Koenig et al. 2002; Schiller et al. 2020b; Kleinert et al. 2022). Thus, some people show substantially higher microstate stability (e.g., an average duration of 120 ms and 8 occurrences per second) than others (e.g., an average duration of 40 ms and 25 occurrences per second). Although research on EEG microstates has increased exponentially in recent years (e.g., da Cruz et al. 2020; de Bock et al. 2020; Schiller et al. 2020a, b; Zanesco et al. 2020; Abreu et al. 2021; Pal et al. 2021; Poskanzer et al. 2021; Kleinert et al. 2022), most studies focused on abnormal microstate characteristics of specific microstate types in clinical populations (for an overview, see Michel and Koenig 2018). Currently, little is known about the functional significance of microstate stability in healthy populations. Here, we examined the hypothesis that the temporal stability of microstates (as indicated by longer durations and fewer occurrences of networks) is positively associated with individual levels of trait aggression, based on the following considerations.

First, resting-state microstate characteristics are prime candidates for neural trait research, which have been associated with the Big 5 personality traits (Zanesco et al. 2020), paranormal belief (Schlegel et al. 2012) and prosocial behavioral preferences (Schiller et al. 2020b). Individual microstate dynamics can be interpreted as “neural signatures” because individuals evidence distinct microstate sequences (Van de Ville et al. 2010) and microstate characteristics are highly reliable (Khanna et al. 2014; Liu et al. 2020; Schiller et al. 2020b) and heritable (da Cruz et al. 2020). Second, common neural sources of resting-state microstates correspond to neural sources of aggression, including the anterior cingulate cortex, the insula, and parts of the prefrontal cortex (Denson et al. 2009; Chester et al. 2014; Dambacher et al. 2015; Repple et al. 2017; Skibsted et al. 2017), brain regions also involved in the regulation of self- and inhibitory control (MacDonald et al. 2000; Allman et al. 2001; Figner et al. 2010; Menon and Uddin 2010). Thus, more effective functioning of these brain regions at rest might contribute to both higher microstate stability as well as increased control over aggressive impulses. Third, a recent study investigated general microstate stability and demonstrated positive associations with trait self-control and a neural index of inhibitory control, and a negative association with risk-taking behavior (Kleinert et al. 2022). As aggression is associated with lower levels of self-control (for reviews, see Baumeister and Boden 1998; Denson et al. 2012; DeWall et al. 2011b), lower levels of inhibitory control (Stucke and Baumeister 2006; Raaijmakers et al. 2008; Pawliczek et al. 2013) and higher levels of risk-taking behavior (for a review, see Kuin et al. 2015), higher microstate stability might indicate lower levels of trait aggression.

To date, only a very few studies have considered possible gender-differences in resting-state microstates (Tomescu et al. 2018; Zanesco et al. 2020). In fact, the majority of studies were based on exclusively male samples (for an overview of studies, see Michel and Koenig 2018). On the other hand, aggression shows large gender-differences (Zell et al. 2015). Factors that contribute to higher levels of aggression in men compared to women include evolutionary adaptation (for reviews, see Campbell 1999; Puts 2016), testosterone availability (for a review, see Archer 2006; for a meta-analysis, see Book et al. 2001), differences in brain-structures (for a review, see Archer 2019), and socialization (Moore and Stuart 2004; Cohn and Zeichner 2006). Furthermore, previous studies found gender-differences in how different facets of aggression are expressed, with females using more indirect/relational aggressive strategies, and males using more direct/physical forms of aggression (Lagerspetz et al. 1988; Cairns et al. 1989; Buss and Perry 1992; Crick and Grotpeter 1995). Based on these findings, we also investigated possible gender-differences in the associations between microstate stability and trait aggression and its facets (i.e., physical aggression, verbal aggression, hostility and anger; Buss and Perry 1992).

## Materials and Methods

### Participants

A total sample of 110 healthy students from Canada (right-handed, native speakers or fluent in English, normal or corrected-to-normal vision and hearing) was recruited for this study. Nine participants were excluded because of bad EEG quality, resulting in a final sample size of *N* = 101 for all statistical analyses (58 women and 43 men; mean age = 19.76 years, range 17–26). Based on an estimated medium effect size of *b* = 0.27 in similar research (Kleinert et al. 2022), an a priori power analysis in G*Power (*α* = 0.05, *power* = 0.85; Faul et al. 2009) resulted in a required sample size of 93 participants. Participants showed normal emotional stability (as measured by the Ten Item Personality Inventory; Gosling et al., 2003) compared to norm values (Gosling et al., 2014; men: *M* = 4.75, *SD* = 0.941; norm values: *M* = 4.61, *SD* = 1.47; women: *M* = 3.99, *SD* = 1.32; norm values: *M* = 4.07, *SD* = 1.46). The study was carried out according to the principles of the Declaration of Helsinki. It was approved by the local university’s ethics committee, and all participants provided informed written consent.

### Procedure

First, participants were seated in an electrically- and noise-shielded cabin, where they were equipped with a 64-electrode EEG system (Brain Products GmbH, Munich, Germany). Next, participants provided demographic information and completed the Aggression Questionnaire along with other scales that are not part of the current study. All materials were in English. Then, we recorded four minutes of resting-state EEG (60 s eyes-open, 60 s eyes-closed, two runs). Alternating eyes-open and eyes-closed periods were used to avoid fatigue in participants and thus achieve more stable EEG recordings (Barry et al. 2007; Schiller et al. 2014, 2019, 2020b; Baumgartner et al. 2019). In line with standard procedures (Damoiseaux et al. 2006; Mantini et al. 2007; for reviews, see Lee et al. 2013; Newson and Thiagarajan 2019), only eyes-closed periods were used for further analyses (max. duration: 120 s). Next, participants completed several computerized tasks that are evaluated elsewhere (Nash et al. 2020, 2021; Kleinert et al. 2022). Note that this study is based on the same data, but uniquely focuses on associations of microstate stability with aggression that have not been previously reported. Finally, participants were compensated for participation with class-credit. All data and code needed to reproduce the results of this work are available in the supplementary material.

### Measurement of Aggression

Participants completed the brief version of the Aggression Questionnaire (AQ; Buss and Perry 1992; Bryant and Smith 2001). The original scale shows good internal consistency (*α* = 0.89) and retest reliability (*r* = 0.80; Buss and Perry 1992), differentiates between participants with higher and lower aggressive behavior (Harris 1997), and is associated with other scales of aggression (Buss and Perry 1992; Harris 1997). The main advantage of the brief version is the superior goodness of fit index of the measurement model (GFI = 0.94) compared to the original scale (GFI = 0.76–0.81). The brief version consists of 12 items, which were rated on 6-point Likert scales ranging from 1 (“not at all characteristic of me”) to 6 (“very much characteristic of me”). It includes the four subscales physical aggression (e.g., “Given enough provocation, I may hit another person”; *α* = 0.72), verbal aggression (e.g., “My friends say that I’m somewhat argumentative”; *α* = 0.78), hostility (e.g., “Other people always seem to get the breaks”; α = 0.75) and anger (e.g., “I have trouble controlling my temper”; *α* = 0.70), which are measured by three items each. Final aggression scores are calculated as the mean of all 12 items (or the mean of the three respective items for subscales), with higher scores indicating more aggression.

### EEG Recording and Preprocessing

We used a 64-electrode EEG system with Ag/AgCI electrodes for electrophysiological recordings of resting-state brain activity (ActiCHamp; Brain Products GmbH, Munich, Germany). Electrodes were positioned according to the 10/10 montage (reference electrode: TP9), and EEG measures were recorded with a sampling-rate of 512 Hz and an online band-pass filter between 0.1 and 100 Hz. Preprocessing of raw EEG data was conducted in the BrainVision Analyzer (version 2.1.0.327; Brain Products GmbH, Munich, Germany). First, we applied a band-pass filter from 1.5 to 20 Hz and a notch filter of 60 Hz to remove any remaining power-line artifacts. Eye movement artifacts were removed using an independent component analysis (ICA). We interpolated channels that were inactive or heavily affected by artifacts (based on expert manual inspections) using neighboring channels. No channels were interpolated in 84 out of 101 participants (83%). On average, 0.267 channels were interpolated (range 0–6). To eliminate any remaining artifacts, we used a semi-automatic artifact-rejection. First, we applied an automatic rejection procedure, in which amplitudes higher than 100 μV or lower than − 100 μV were defined as artifacts. This procedure was followed by an expert manual inspection of the data to reject any remaining artifacts that were not captured by the automatic procedure. Then, EEGs were segmented into eyes-open and eyes-closed periods. Finally, signals were re-derived to average reference and segmented into epochs of two seconds for microstate analysis (eyes-closed periods only).

### EEG Microstate Analysis

A plugin for resting-state microstate analysis by Koenig (2017) was used to quantify individual levels of microstate stability in EEGLAB (Delorme and Makeig 2004). The plugin follows standard procedures (Lehmann et al. 1987; Strik and Lehmann 1993; Wackermann et al. 1993). First, artifact-free, eyes-closed EEG signals from all 64 electrodes were used to extract electric potential field maps at timepoints of maximum global field power (GFP). Only using maps from GFP-peaks results in an optimal signal-to-noise ratio (Koenig et al. 2002). Second, the four most predominant individual microstate-maps were identified using an atomize-agglomerate hierarchical clustering procedure (AAHC; Murray et al. 2008; Michel et al. 2009). Third, individual maps were submitted to another cluster analysis yielding grand-mean microstate maps across subjects, which were sorted to follow the standard order of A, B, C, D (Koenig et al. 2002). Note that the four grand-mean microstate maps closely resemble the four prototypical resting-state microstate types (see Table 1; for reviews, see Khanna et al. 2015; Michel and Koenig 2018). Fourth, mean microstate maps of each subject were assigned to one of the four prototypical microstate types based on spatial correlations with grand-mean maps. Fifth, continuous sequences of microstate maps were obtained by assigning each subject’s electric potential field maps (see step 1) to the best fitting mean microstate map. These individual sequences were used to obtain average durations of all four microstate types in milliseconds (*duration A–D*) and the average number of occurrences of all four microstate types per second (*occurrence A–D*). *Mean duration* refers to the average duration across all four microstate types A–D, and *mean occurrence* refers to the average number of occurrences of all four microstate types A–D per second. Note that mean duration and mean occurrence are negatively related to each other, as longer durations naturally predict fewer occurrences of microstates. Therefore, we use the term *microstate stability* to describe the overall, microstate type-independent tendency of individuals to show longer durations and fewer occurrences of resting-state microstates.

**Table 1:**
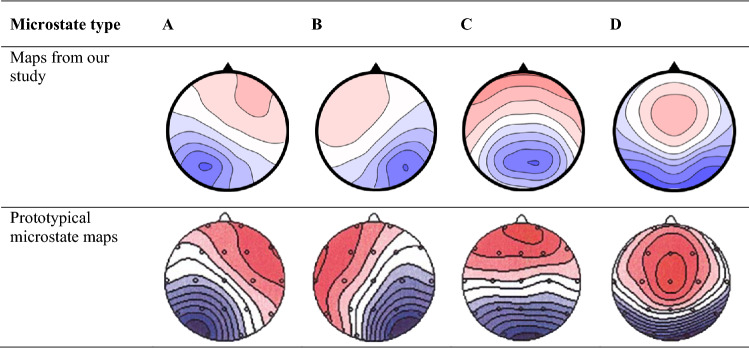
Grand-mean microstates maps

*N* = 101. Top: Grand-mean microstate maps from our study. Bottom: Prototypical grand-mean microstate maps (Koenig et al. 2002). Note that microstate maps of our study closely resemble the four prototypical resting-state microstate maps

### Statistical Analysis

We first investigated the overall tendency of individuals for higher or lower microstate stability by comparing a basic model of microstate duration (and microstate occurrence) across microstate types (data in long format) with a mixed model of microstate duration (and microstate occurrence) including a random intercept across participants (also see Kleinert et al. 2022). If the models including a random intercept would show significantly higher model fits, this would confirm substantial intercorrelations of durations (and occurrences) within individuals. Additionally, we analyzed effect sizes of these intercorrelations using intra-class correlations (ICC). To investigate our main hypotheses, we regressed aggression on the average duration (and average occurrence) of resting-state microstates (data in wide format). We then tested if gender would moderate associations between aggression and average microstate duration (and average microstate occurrence) in Process (Hayes 2017). Moderation analysis tests whether the magnitude or direction of an association between two variables depends on a third variable (i.e., the moderator) by including the interaction between the independent variable and the moderator in the regression term. To analyze any gender-differences in detail, we calculated regression analyses separately in men and women. Finally, we repeated all previous analyses separately for the four subscales of our aggression scale to analyze specific associations of microstate stability with hostility, physicality, verbal aggression, and anger.

## Results

### Resting-State Microstates

The average EEG time available for microstate analyses was 105.64 s (range 50.94–112.70 s). Consistent with previous studies, the four prototypical microstate types explained an average of 74.71% of variance in EEG signals (range 55.93–84.43%; see Table S1 in the supplementary material for detailed descriptive statistics). Supporting the idea of a stable individual tendency for higher or lower microstate stability across microstate types, a mixed model of general microstate duration including a random intercept across participants resulted in a higher model fit compared to a simple model of general microstate duration (*p* < 0.001). The same applied for general microstate occurrence (*p* < 0.001). Intraclass correlations confirmed substantial intercorrelations among durations and occurrences of the four microstate types (durations: *ICC* = 0.747, occurrences: *ICC* = 0.729). These results confirm the usefulness of analyzing the mean duration (and the mean occurrence) of resting-state microstates.

### Associations of Microstate Stability and Aggression

**Fig. 1:**
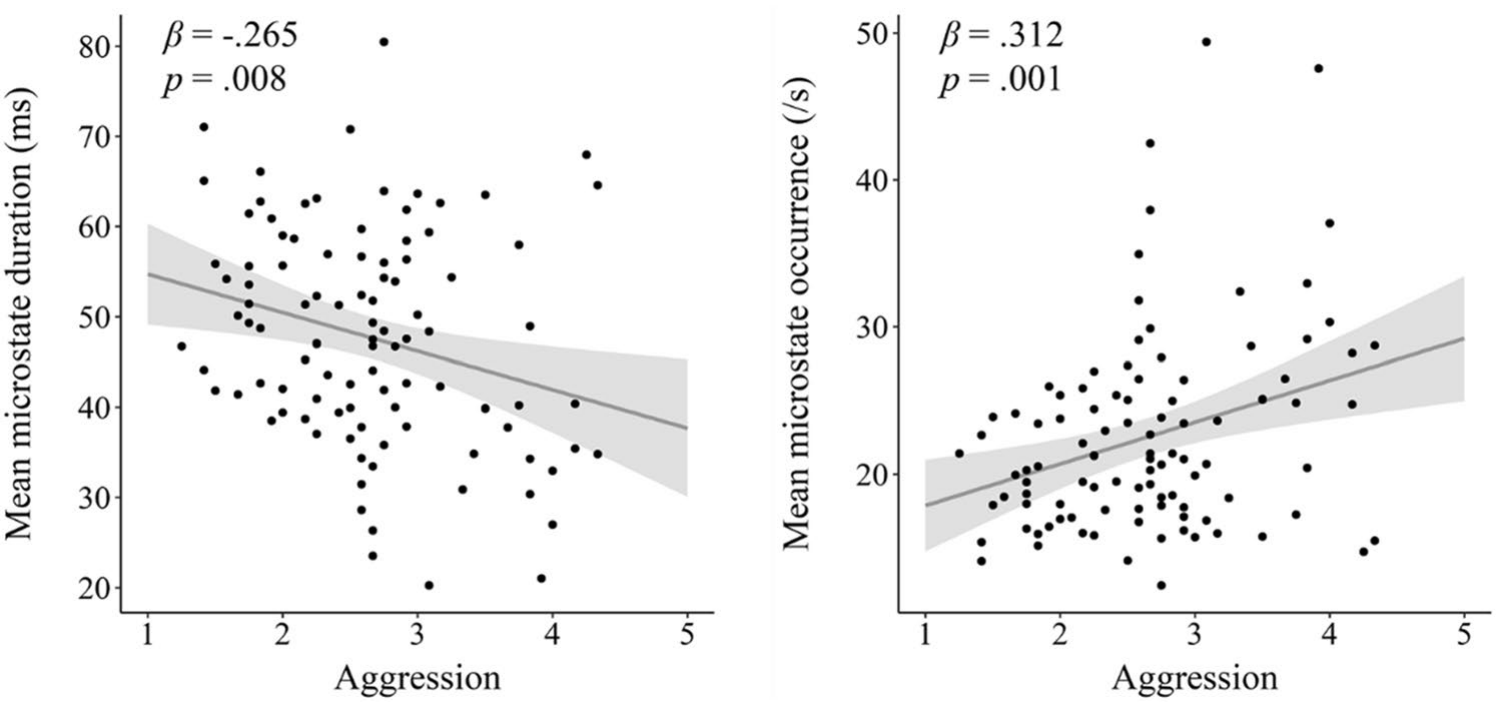
Associations of mean microstate duration and mean microstate occurrence with aggression. Left: Scatterplot showing the association of mean microstate duration with aggression. Right: Scatterplot showing the association of mean microstate occurrence with aggression. Both plots include 95% confidence intervals, standardized regression coefficients and p-values. Taken together, these results suggest that microstate stability indicates lower levels of aggression

As hypothesized, mean microstate duration was negatively associated with aggression (*β* = − 0.265, *p* = 0.008, *R*^*2*^ = 0.070; see Fig. 1 and Table 2). The subscales physical aggression, verbal aggression and hostility contributed to this effect, but not the subscale anger (see Table 2). Furthermore, mean microstate occurrence was positively associated with aggression (*β* = 0.312, *p* = 0.001, *R*^*2*^ = 0.098; see Fig. 1 and Table 2). Again, the subscales physical aggression, verbal aggression and hostility contributed to this effect, but not the subscale anger (see Table 2). In sum, these results suggest that lower microstate stability is associated with aggression. Similar to Kleinert and colleagues (2022), these effects were stable across all four prototypical microstate types (see Table S2 in the supplementary material).

**Table 2:** Gender differences in associations of microstate stability with aggression

Dependent variable	Predictor Mean microstate duration			Moderator model (interaction with gender; interaction term)						
	N	Male	Female	Int. b	t(97)	Int. p	LLCI	ULCI	R^2^	R^2^-change
Total score: aggression	− 0.265**	− 0.471**	− 0.141	0.409	2.03	0.045	0.009	0.809	0.114	0.038
Subscale: physical aggression	− 0.252*	− 0.443**	− 0.093	0.424	2.11	0.038	0.025	0.823	0.120	0.040
Subscale: verbal aggression	− 0.261**	− 0.241	− 0.276*	0.050	0.243	0.808	− 0.360	0.460	0.069	0.001
Subscale: hostility	− 0.207*	− 0.337*	− 0.171	0.185	0.900	0.371	− 0.223	0.591	0.083	0.008
Subscale: anger	− 0.034	− 0.378*	0.139	0.537	2.65	0.010	0.134	0.941	0.101	0.065

Dependent variable	Predictor Mean microstate occurrence			Moderator model (interaction with gender; interaction term)						
	N	Male	Female	Int. b	t(97)	Int. p	LLCI	ULCI	R^2^	R^2^change
Total score: aggression	0.312**	0.437**	0.226^†^	− 0.239	− 1.23	0.223	− 0.624	0.147	0.117	0.014
Subscale: physical aggression	0.287**	0.404**	0.175	− 0.257	− 1.32	0.189	− 0.643	0.129	0.116	0.016
Subscale: verbal aggression	0.217*	0.174	0.249^†^	0.038	0.188	0.852	− 0.362	0.438	0.048	< 0.001
Subscale: hostility	0.276**	0.354*	0.257^†^	− 0.073	− 0.376	0.708	− 0.460	0.314	0.110	0.001
Subscale: anger	0.117	0.381*	− 0.037	− 0.411	− 2.09	0.040	− 0.802	− 0.020	0.091	0.041

*N* = 101, *N*_*male*_ = 43, *N*_*female*_ = 58. Left: Shown are standardized regression coefficients (β, two-sided tests, alpha level = 0.05) illustrating associations of microstate stability (i.e., longer microstate duration and fewer microstate occurrences) with aggression scores. Right: Shown are moderator models (whole sample; two-sided tests, alpha level = 0.05) illustrating interaction effects of the previous associations with gender. Across genders, higher microstate stability indicates lower levels of aggression, due to negative associations of microstate duration, and positive associations of microstate occurrence with physical, verbal and hostile aggression. Looking at gender-differences, men show stronger negative associations of microstate stability with aggression than women. This effect is due to stronger negative associations of microstate duration with physical aggression and anger, and stronger positive associations of microstate occurrence with physical aggression, hostility and anger in men compared to women

*Int. b* coefficient of interaction effect, *t(df)* t value (and degrees of freedom) of interaction effect, *Int. p* p value of interaction effect, *LLCI* lower limit of confidence interval, *ULCI* upper limit of confidence interval, *R*^*2*^ variance explained, *R*^*2*^*-change* Change in variance explained by introducing gender as a moderator

^†^p < 0.10, *p < 0.05, **p < 0.01, ***p < 0.001

### Gender Differences

**Fig. 2:**
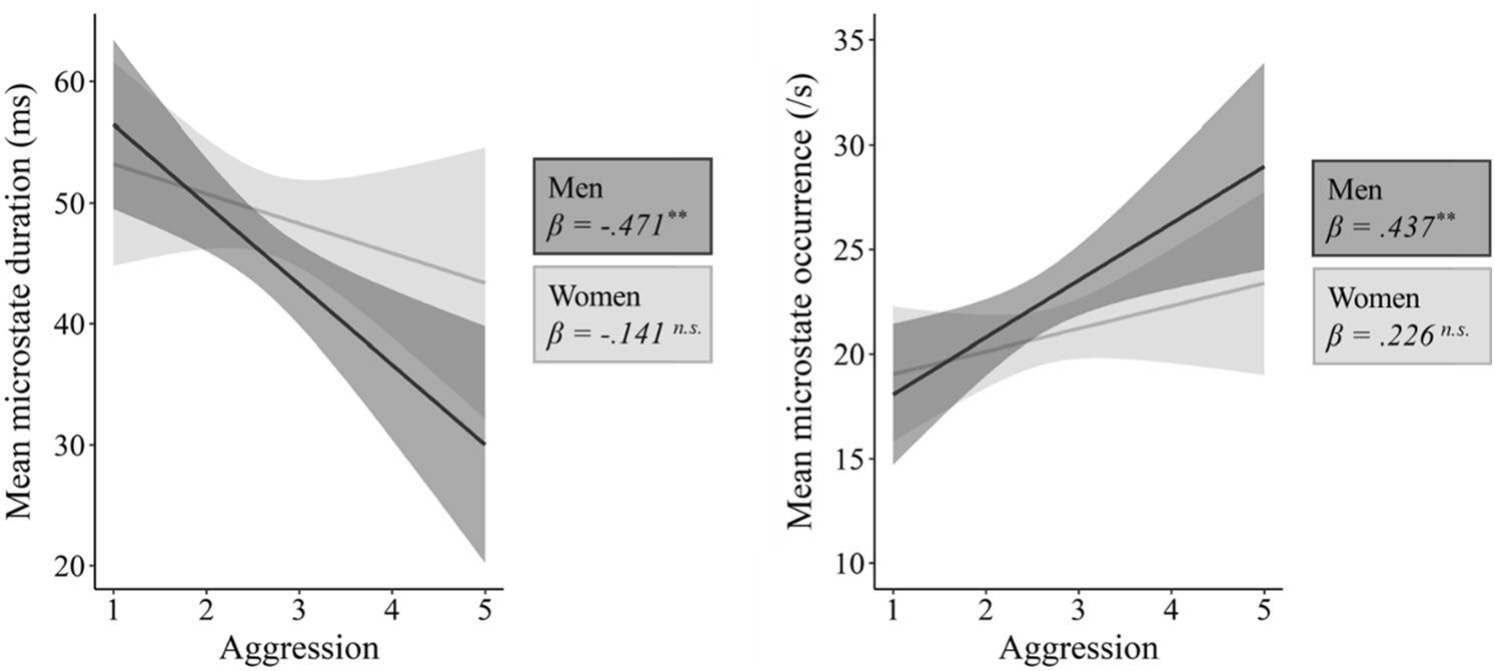
Gender differences in associations of microstate stability with aggression. Left: Interaction plot illustrating a stronger negative association of mean microstate duration with aggression in men compared to women (interaction effect: *p* = 0.045; *β*_*men*_ = − 0.471*, p*_*men*_ = 0.001 vs *β*_*women*_ = − 0.141, *p*_*women*_ = 0.290). Right: Interaction plot illustrating a stronger positive association of mean microstate occurrence with aggression in men compared to women (interaction effect: *p* = 0.223; *β*_*men*_ = 0.437, *p*_*men*_ = 0.003 vs *β*_*women*_ = 0.226, *p*_*women*_ = 0.088). Gender moderated the association of mean microstate duration and aggression, but not the association of mean microstate occurrence and aggression. Both plots include 95% confidence intervals and standardized regression coefficients (**p < 0.01; *n.s.* not significant). In sum, the association of aggression with microstate stability was stronger in men compared to women

Using model 1 in the process-macro for moderation analyses (Hayes 2017), we found that the negative association of aggression and mean microstate duration is moderated by gender, with men showing a larger negative association than women (interaction effect: *p* = 0.045; *β*_*men*_ = − 0.471, *p*_*men*_ = 0.001 vs *β*_*women*_ = − 0.141, *p*_*women*_ = 0.290; see Fig. 2 and Table 2; for separate analyses for each microstate type see Table S2 in the supplementary material). The subscales physical aggression and anger contributed to this effect, but not the subscales verbal aggression and hostility (see Table 2). These results demonstrate that the negative association of mean microstate duration and aggression applies primarily to men, and only to a lesser extent to women. Furthermore, we found that the positive association of aggression and mean microstate occurrence is not moderated by gender (interaction effect: *p* = 0.223; *β*_*men*_ = 0.437, *p*_*men*_ = 0.003 vs *β*_*women*_ = 0.226, *p*_*women*_ = 0.088). However, there were significant associations of mean microstate occurrence with aggression (and with the subscales physical aggression, hostility and anger) in men, but not in women. Also note that gender moderated the association of the subscale anger with mean microstate occurrence, with men showing a larger positive association than women.

## Discussion

In line with our main hypothesis, shorter durations and more occurrences of microstates, indicating lower microstate stability, were observed in individuals with higher levels of trait aggression. This finding complements previous associations of microstate stability with self-control, inhibitory control and risk-taking (Kleinert et al. 2022). Broadly, our results provide further support for the notion that microstate stability at rest constitutes an important neural source of individual heterogeneity. More specifically, our results illustrate that trait aggression is predicted by the basic temporal organization of the resting brain. Brain areas associated with both trait aggression and resting-state microstates significantly overlap, including the anterior cingulate cortex, the insula, and parts of the prefrontal cortex (MacDonald et al. 2000; Allman et al. 2001; Denson et al. 2009; Figner et al. 2010; Menon and Uddin 2010; Chester et al. 2014; Dambacher et al. 2015; Custo et al. 2017; Milz et al. 2017; Repple et al. 2017; Skibsted et al. 2017). Possibly, more effective functioning of these neural sources at rest contributes to a higher microstate stability as well as a higher capacity to control aggressive impulses, and thus predisposes for lower levels of trait aggression. Indeed, a recent study found that the functional connectivity of resting networks, including both the ACC and the insula, is related to aggressive impulses in combat veterans (Varkevisser et al. 2017). Future studies are needed to further examine this mechanistic framework, e.g., by investigating how microstate stability at rest is related to task-related brain activity associated with aggression, and, importantly, if it causally predicts actual subsequent aggressive behavior.

Based on research demonstrating higher levels of aggression in men compared to women (Zell et al. 2015), as well as gender effects in the expression of aggression (Lagerspetz et al. 1988; Cairns et al. 1989; Buss and Perry 1992; Crick and Grotpeter 1995), we analyzed possible gender-differences in the association between microstate stability and trait aggression. Overall, we found that the association of microstate stability and trait aggression was stronger in men compared to women. To the best of our knowledge, we note that this is the first study to show gender-differences in the functional significance of resting-state microstates. More specifically, our results suggest that trait aggression might be represented by a lower temporal stability of resting networks in men, whereas aggression in women might primarily result from more complex, situational neural mechanisms. This notion is in line with research on the preferred usage of direct forms of aggression in males, and more complex aggressive strategies, such as relational or indirect aggression, in females (Lagerspetz et al. 1988; Crick and Grotpeter 1995). Investigating different facets of aggression, we found that gender differences occurred due to higher associations of microstate stability with physical aggression and anger in men compared to women, but not with verbal aggression or hostility. Physical aggression can be characterized as a typical male way to express aggression (Cairns et al. 1989; item example: "Given enough provocation, I may hit another person"; Buss and Perry 1992) and anger is operationalized in the Aggression Questionnaire as a non-cognitive trait reflecting impulsivity (correlation of anger with impulsivity: *r* = 0.42; item example: “I flare up quickly but get over it quickly”; Buss and Perry 1992). On the other hand, verbal aggression requires higher order brain functions to engage in language processing (item example: “I can’t help getting into arguments when people disagree with me”; Buss and Perry 1992), and hostility represents a ruminative, cognitively driven trait (item example: “Other people always seem to get the breaks”; Buss and Perry 1992). Thus, our results support the assumption of an easily accessible, microstate driven pathway to aggression in men, which is less pronounced in women (especially concerning physical and impulsive aggression).

In summary, this study provides novel insights into the largely unknown functional significance of resting-state microstates and indicates for the first time that this functional significance might differ between men and women. Based on this study and recent projects (Schiller et al. 2020b; Nash et al. 2022; Kleinert et al. 2022), resting-state microstate characteristics constitute a unique and predictive measure in neural trait research. In particular, resting-state microstate stability provides a task-independent window into individual heterogeneity in fundamental neural network dynamics, and this neural heterogeneity has been associated with key differences at the trait level. This has opened up numerous avenues for important future research. For example, studies are needed to further evaluate origins, mechanics, and functions of microstate stability. More research is also needed to shed light on the exact causal mechanisms that underly associations of resting-state microstates with human traits. Finally, larger and more diverse samples are required to enable investigations of differences in the functional significance of microstates regarding gender, age, culture, education and economic background.

### Supplementary Information

Below is the link to the electronic supplementary material.

Supplementary file1 (DOCX 30 kb)

## Data Availability

All data and code needed to reproduce the results of this work are freely available online in the OSF repository (https://osf.io/cen3s).
