# Supplementary material for: Trait Aggression is Reflected by a Lower Temporal Stability of EEG Resting Networks
Source: Brain Topogr. 2022 Nov 18;37(4):514–23. doi: 10.1007/s10548-022-00929-6 (PMC11199292; doi:10.1007/s10548-022-00929-6)
Supplement: Supplementary file 1 — Supplementary file1 (DOCX 30 kb) [file 10548_2022_929_MOESM1_ESM.docx]

**Supplementary Material**

Trait aggression is reflected by a lower temporal stability of EEG resting networks

Running title: EEG microstates and aggression

Tobias Kleinert^1,2^, Kyle Nash^1^

1. Department of Psychology, University of Alberta, T6G 2E9 Edmonton, AB, Canada
2. Department of Ergonomics, Leibniz Research Centre for Working Environment and Human Factors, 44139 Dortmund, Germany

Corresponding author:

Dr. Tobias Kleinert

Mail: kleinert@ifado.de

Phone: +49 15739645375

Leibniz Research Centre for Working Environment and Human Factors

Ardeystr. 67, 44139 Dortmund, Germany

**Table S1** Descriptive statistics

| **Variable** | **Min** | **Max** | **Mean** | **SD** |
| --- | --- | --- | --- | --- |
| **Measures of aggression** | | | | |
| Total score: Aggression | 1.25 | 5.33 | 2.65 | .746 |
| Subscale: Physical aggression | 1.00 | 4.33 | 2.06 | .977 |
| Subscale: Verbal aggression | 1.00 | 6.00 | 3.14 | 1.14 |
| Subscale: Hostility | 1.00 | 5.33 | 2.78 | 1.02 |
| Subscale: Anger | 1.00 | 6.00 | 2.63 | 1.04 |
| **Characteristics of resting-state microstates** | | | | |
| Duration microstate A | 20.63 | 84.49 | 47.20 | 13.10 |
| Duration microstate B | 13.03 | 76.17 | 44.86 | 11.50 |
| Duration microstate C | 15.68 | 78.73 | 48.63 | 13.34 |
| Duration microstate D | 20.25 | 85.15 | 48.03 | 14.39 |
| Mean duration | 20.25 | 80.47 | 47.69 | 11.99 |
| Occurrence microstate A | 3.14 | 15.47 | 5.63 | 1.93 |
| Occurrence microstate B | 2.37 | 11.59 | 5.29 | 1.75 |
| Occurrence microstate C | 2.92 | 15.25 | 5.86 | 1.84 |
| Occurrence microstate D | 3.25 | 15.26 | 5.76 | 2.01 |
| Mean occurrence | 12.43 | 49.39 | 22.54 | 6.77 |

*N* = 101. Min = minimum value, Max = maximum value, Mean = mean value, SD = standard deviation.

**Table S2** Associations of microstate stability with aggression scores for each microstate type and gender

|  | **Predictor: Durations of each microstate type** | | | | | | | | | | | |
| --- | --- | --- | --- | --- | --- | --- | --- | --- | --- | --- | --- | --- |
|  | **Duration A** | | | **Duration B** | | | **Duration C** | | | **Duration D** | | |
| Dep**endent variable** | **N** | **male** | **fem.** | **N** | **male** | **fem.** | **N** | **male** | **fem.** | **N** | **male** | **fem.** |
| Total score: Aggression | -.190^†^ | -.325^*^ | -.105 | -.260^**^ | -.407^**^ | -.179 | -.255^*^ | -.435^**^ | -.140 | -.248^*^ | -.487^***^ | -.096 |
| Subscale: Physical aggression | -.198^*^ | -.379^*^ | -.028 | -.272^**^ | -.421^**^ | -.135 | -.224^*^ | -.370^*^ | -.102 | -.225^*^ | -.405^**^ | -.076 |
| Subscale: Verbal aggression | -.199^*^ | -.141 | -.242^†^ | -.209^*^ | -.123 | -.270^*^ | -.246^*^ | -.284^†^ | -.215 | -.271^**^ | -.279^†^ | -.265^*^ |
| Subscale: Hostility | -.186^†^ | -.219 | -.208 | -.165^†^ | -.282^†^ | -.145 | -.227^*^ | -.307^*^ | -.207 | -.166^†^ | -.362^*^ | -.081 |
| Subscale: Anger | .042 | -.231 | .187 | -.098 | -.404^**^ | .040 | -.029 | -.315^*^ | .127 | -.039 | -.394^**^ | .151 |
|  | **Predictor: Occurrences of each microstate type** | | | | | | | | | | | |
|  | **Occurrence A** | | | **Occurrence B** | | | **Occurrence C** | | | **Occurrence D** | | |
| **Dependent variable** | **N** | **male** | **fem.** | **N** | **male** | **fem.** | **N** | **male** | **fem.** | **N** | **male** | **fem.** |
| Total score: Aggression | .281^**^ | .341^*^ | .237^†^ | .256^**^ | .399^**^ | .164 | .291^**^ | .402^**^ | .196 | .293^**^ | .366^*^ | .240^†^ |
| Subscale: Physical aggression | .276^**^ | .347^*^ | .196 | .236^*^ | .336^*^ | .146 | .267^**^ | .385^*^ | .111 | .252^*^ | .320^*^ | .190 |
| Subscale: Verbal aggression | .152 | .069 | .227^†^ | .230^*^ | .276^†^ | .191 | .251^*^ | .171 | .336^**^ | .154 | .105 | .194 |
| Subscale: Hostility | .255^*^ | .335^*^ | .225^†^ | .233^*^ | .266^†^ | .241^†^ | .183^†^ | .274^†^ | .155 | .315^**^ | .339^*^ | .320^*^ |
| Subscale: Anger | .130 | .292^†^ | .031 | .031 | .289^†^ | -.109 | .127 | .373^*^ | -.049 | .125 | .349^*^ | -.017 |

*N* = 101, *N_male_* = 42, *N_female_* = 58. ^†^ = p < .10, ^*^ = p < .05, ^**^ = p < .01, ^***^ = p < .001. Standardized regression coefficients (β, two-sided tests, alpha level = .05) showing associations of microstate duration and microstate occurrence with aggression and its subscales for each microstate type and gender (male and female). Note that in the whole sample (N), 19/20 associations of microstate durations with aggression scores are negative, and 20/20 associations of microstate occurrences with aggression scores are positive. Overall, men tend to show stronger negative associations of microstate durations with aggression scores and stronger positive associations of microstate occurrences with aggression scores compared to women.
